# Structural barriers in access to medical marijuana in the USA—a systematic review protocol

**DOI:** 10.1186/s13643-017-0541-4

**Published:** 2017-08-07

**Authors:** Celina I. Valencia, Ibitola O. Asaolu, John E. Ehiri, Cecilia Rosales

**Affiliations:** 1 Department of Community, Environment, and Policy, Mel and Enid Zuckerman College of Public Health, University of Arizona, Tucson, AZ USA; 2 Department of Health Promotion Sciences, Mel and Enid Zuckerman College of Public Health, University of Arizona, Tucson, AZ USA; 3 Division of Public Health Practice and Translational Science, Mel and Enid Zuckerman College of Public Health, University of Arizona, Phoenix, AZ USA

## Abstract

**Background:**

There are 43 state medical marijuana programs in the USA, yet limited evidence is available on the demographic characteristics of the patient population accessing these programs. Moreover, insights into the social and structural barriers that inform patients’ success in accessing medical marijuana are limited. A current gap in the scientific literature exists regarding generalizable data on the social, cultural, and structural mechanisms that hinder access to medical marijuana among qualifying patients. The goal of this systematic review, therefore, is to identify the aforementioned mechanisms that inform disparities in access to medical marijuana in the USA.

**Methods:**

This scoping review protocol outlines the proposed study design for the systematic review and evaluation of peer-reviewed scientific literature on structural barriers to medical marijuana access. The protocol follows the guidelines set forth by the Preferred Reporting Items for Systematic review and Meta-Analysis Protocols (PRISMA-P) checklist.

**Discussion:**

The overarching goal of this study is to rigorously evaluate the existing peer-reviewed data on access to medical marijuana in the USA. Income, ethnic background, stigma, and physician preferences have been posited as the primary structural barriers influencing medical marijuana patient population demographics in the USA. Identification of structural barriers to accessing medical marijuana provides a framework for future policies and programs. Evidence-based policies and programs for increasing medical marijuana access help minimize the disparity of access among qualifying patients.

## Background

The USA is witnessing an increase in marijuana use. In 2012, an estimated 7.6 million individuals used marijuana daily or almost daily, representing a considerable increase in marijuana use from 5.1 million individuals in 2007 [1]. The Pew Research Center [2] reported that 30% of marijuana use in 2012 was for the purpose of disease-related symptom treatment, which the National Institutes of Health (NIH) classify as medical marijuana. Marijuana is used for medicinal purposes because it contains tetrahydrocannabinol, a cannabinoid that stimulates appetite and manages pain and nausea [3]. The medicinal effects of marijuana fall under the scope of medical marijuana laws (MMLs) that regulate the dispensation of marijuana for medicinal purposes. To date in the USA and US territories, active medical marijuana programs are present in 43 states, the District of Columbia, Guam, and Puerto Rico [4]. MMLs allow individuals with qualifying medical conditions to receive physician authorization to participate in these jurisdictions’ medical marijuana programs [5].

The variation of MMLs across the USA largely dictates patterns of access, prescription, dispensation, and permissible use of medical marijuana. Additional factors such as income, race/ethnic background, stigma, and physician characteristics have emerged as mechanisms that foster an incipient area of disparity: medical marijuana accessibility. Access to medication has been discussed widely as a manifestation of structural violence in literature pertaining to social determinants of health [6, 7]. Structural violence in the form of constrained access to medication impedes individuals from obtaining the necessary provisions for their health and well-being. This review will focus on the interactions among the ways in which MML constrains access to medical marijuana among traditionally vulnerable and underserved populations. The following section provides a brief discussion of what is currently known regarding structural barriers to medical marijuana use.

### Race or ethnic background

Medical marijuana use is predominant among white Americans, and less likely among Latino, Asian, and foreign-born individuals [8, 9]. There are few insights into why racial minorities in the USA have lower prevalence of medical marijuana use. Reinarman et al. [10] attribute the underrepresentation of Latinos’ use of medical marijuana to being a consequence of exclusionary immigration policies that hinder their participation in governmental programs [11, 10]. Due to the fear of immigration, certain ethnic minorities may not seek medical marijuana use despite their qualifying health conditions [10]. The association between race/ethnicity and levels of disposable income is cited as an underlying reason for why ethnic minorities in general might be less likely to use medical marijuana [12, 10]. Marijuana possession remains a federal crime, and ethnic minorities continue to be charged significantly more frequently than white males for marijuana-related offenses [13]. Therefore, the higher likelihood of ethnic minorities, such as Latinos and African Americans, being targeted by law enforcement or facing legal consequences for involvement with marijuana could also deter their use of medical marijuana [13].

### Socioeconomic background

Medical marijuana use is more common among individuals who are employed, have health insurance, and earn high incomes. For example, California residents earning over $60,000 are most likely to use medical marijuana [8]. Similarly, a major proportion (64.8%) of medical marijuana patients in medical marijuana assessment clinics across California reported being employed while almost three quarters (73.4%) of them had private health insurance [10].

Issuance of medical marijuana cards across states with MMLs usually has a financial cost. In certain cases, states may provide a cost subsidy for medical marijuana users who also qualify for federal assistance programs such as Medicaid or the Supplemental Nutrition Assistance Program (SNAP). In Arizona, for instance, the standard fee is $150.00 for both initial and renewal registrations for the state’s medical marijuana program; registrants must also have a valid referral from an authorized medical provider [14]. Neither the cost of receiving a medical provider referral nor the state registration fee is covered by health insurance. If the qualifying patient is currently participating in SNAP, they may register at a subsidized rate of $75.00 [14]. Thus, without sufficient disposable income, health insurance, or subsidies, individuals with qualifying medical conditions may be unable to participate in the medical marijuana program offered by their jurisdiction.

### Stigma

Stigma associated with possessing and using marijuana could further deter individuals from considering medical marijuana. For instance, the fear of labels such as “junkie” or “stoner” may dissuade patients with qualifying medical conditions from discussing medical marijuana use with their healthcare provider [15]. This fear could also prevent qualifying patients from visiting a licensed dispensary to purchase marijuana [15]. Statutes legalizing medical marijuana could reduce the stigma associated medical marijuana use due to the sheer increase in its availability (Pacula et al. [16]) and resulting social acceptability [5].

### Physicians

Another predictor of medical marijuana initiation and continuous use is physician attitudes. Under many state medical marijuana programs, a licensed physician must certify that a patient has a qualifying medical condition before that patient can access medical marijuana [3]. Depending on their source of information, physicians can be ambivalent about making these recommendations. In fact, many physicians cite the physical and mental health risks of medical marijuana as deterrents to recommending qualified patients [3].

Two further significant drawbacks to recommending medical marijuana are its classification as a “schedule I illegal drug” by the U.S. Drug Enforcement Administration and its unapproved status by the Federal Drug Administration [3]. The potential for physicians to resist recommending medical marijuana use is also identified in the literature. For example, 39% of specialists and 34% of primary care physicians in Delaware reported being “very unlikely” to authorize eligible patients for medical marijuana [17]. In addition, Satterlund et al. [15] found that patients do not initiate medical marijuana-related conversations with their primary care provider because of anticipated negative responses.

No previous systematic reviews have been conducted to assess the relative importance of the afore-discussed factors in influencing access to medical marijuana in the USA. Thus, the objective of this review is to summarize and critically appraise information regarding structural barriers to medical marijuana use in the USA (Arksey and O’Malley, [18]). The review will draw from the literature on the social determinants of health to identify established barriers of access to medication [7]. The structural barriers to access framework builds on the acknowledgment of structural violence as an impediment to health provisions [6]. Structural barriers in access to medical marijuana use in the USA have not been comprehensively analyzed, creating a gap in the literature. As medical marijuana programs proliferate across the USA, it is imperative to identify mechanisms driving the disparities to accessing this form of treatment. This review seeks to generate scientific evidence to fill the current gap in the literature, inform evidence-based policies and programs, and stimulate collective public health discourse on medical marijuana. Tackling structural barriers to medical marijuana access encourages equity across all adult patient population and aligns with the fundamental pursuit of public health research and practice.

## Methods

### Inclusion criteria

#### Type of studies

The primary outcome identified by qualifying studies must be medical marijuana or medical cannabis. All research designs and studies using either qualitative or quantitative methodologies are eligible for inclusion in this review. Included studies must be peer-reviewed scientific investigations of social, economic, and/or cultural processes in the USA which are likely to be structural barriers to accessing medical marijuana. Excluded from this review are studies that focused on addiction or recreational marijuana use, as they fall outside the scope of legal medical marijuana programs in the USA.

#### Type of participants

Only studies limited to populations residing in the USA and its territories will be considered in this review. This geographical restriction relates to the review’s focus on access disparities as a result of social, economic, and/or cultural processes in the USA. Study populations must also meet all legal requirements set forth by state or jurisdiction medical marijuana programs. Since the objective of this review is to identify sociodemographic factors that may increase disparity in medical marijuana use and access, it is vital to allow for an in-depth consideration of all sociodemographic information that emerges from the literature. Therefore, this review will not place restrictions on demographics or social factors contained within any study’s population.

#### Type of outcome measures

The primary outcome of this study is to identify the social, economic, and cultural processes in the USA, which could serve as structural barriers to accessing medical marijuana programs. Social determinants of health—including education, employment, race, and healthcare—will be emphasized.

#### Search methods

To identify studies that reported on social, economic, and cultural processes that could serve as structural barriers to accessing medical marijuana programs in the USA, the following six electronic databases will be searched: PubMed, Embase, CINAHL, PsycINFO, EBSCO, and Web of Science. Since the review will focus solely on studies conducted in the USA, we anticipate that articles found in this search will be published in English. The searches will be coordinated by the University of Arizona College of Public Health librarian. Details of the terms and concepts that will be searched are presented in Appendix 2.

The study did not require Institutional Review Board approval as it is a secondary analysis of peer-reviewed literature; no human subjects are involved. The study adheres to the conventions set forth in the PRISMA-P guidelines (Appendix 1).

### Data collection and analysis

#### Selection of studies

Two reviewers (CIV and IOA) will be assigned to conduct independent screening of all eligible articles. Each reviewer will assess article abstracts against the aforementioned inclusion and exclusion criteria in this protocol. Following this initial review, the reviewers will meet to compare their determinations.

Furthermore, reviewers will confirm agreement of all article categorizations. In instances where the reviewers disagree, they will discuss their reasons for differing categorizations. If reviewers are unable to come to agreement, the matter will be deferred to the senior members (JEE and CR) of the research team for their executive decision. Articles with unavailable abstracts will be requested through the University of Arizona’s Interlibrary Loan (ILL) program. If the article is not available via ILL, the research team will contact the article’s corresponding author for an electronic and/or hard copy of the article. Articles that cannot be obtained through either of these mechanisms will be excluded from the study.

The second review step will consist of the reviewers independently screening full article manuscripts. Afterwards, the two reviewers will meet to compare determinations for inclusion or exclusion. The reviewers will discuss article categorizations based on independent review and submit included studies to senior researchers for final assessment. Subsequently, the data extraction and management phases of the review will commence.

EndNote will be used as the reference management software. Microsoft Excel spreadsheets will be used to manage inclusion and exclusion during the initial review phase.

#### Assessment of publication and reporting bias

Publication bias, a form of reporting bias, arises when the dissemination of research findings is influenced by the nature and direction of results [19]. To address publication bias in the review, we will conduct an exhaustive search of major electronic databases in order to identify all potentially eligible studies. Further, to reduce bias towards reports that are abstracted in mainstream databases, the search will be extended to include Google, Google Scholar, and the gray literature. To obtain information on completed and ongoing studies, we will hand-search reference lists of identified articles, and contact investigators and organizations whose research or programs relate to access to medical marijuana. It is common knowledge that studies which yield statistically significant results are more likely to be reported than those with non-significant findings. To address reporting bias in this review, we will, where possible, compare and discuss the strength, as well as pattern of evidence from studies published in mainstream journals to those published in the gray literature. Where necessary, we will approach authors for full data from their results so we can assess and come to informed decision regarding the nature and strength of the evidence reported by the authors.

#### Assessment of study quality

We will assess the quality of case-control and cohort studies using the Newcastle Ottawa Scale [20]. For case-control studies, we will assess the adequacy of case and control definition, representativeness of the cases, whether controls were derived from the same population as cases, comparability of cases and controls on the basis of design and analyses, and ascertainment of exposure and non-response rates. For cohort studies, we will assess the representativeness of the exposed cohort in the study setting, the selection of the non-exposed cohort, ascertainment of exposure, demonstration that the outcome of interest was not present at start of the study, comparability of cohorts on the basis of design and analyses, outcome assessment, and adequacy of follow-up [20]. After this assessment process, we will assign a composite quality score of 0 to 9. Studies that score less than 6 will be judged to be of low quality.

For cross-sectional studies, we will use the guidelines for critical appraisal developed by the National Collaborating Center for Environmental Health [21]. We will assess representativeness of study participants; methods for ascertaining exposure; comparability of exposure groups in terms of age, sex, socio-economic status, and non-response bias; determination and validation of outcomes; internal validity; and how confounding factors were assessed and addressed. After this assessment process, we will assign a composite quality score of 0 to 4. Two reviewers (IOA and CIV) will assess study quality and reach a consensus score for each included study.

Qualitative studies will be appraised using the checklist for appraisal of qualitative research developed by the Critical Appraisal Skills Programme (CASP), Oxford, UK [22]. The CASP tool assesses the methodological strength of qualitative studies and consists of ten questions that are designed to evaluate conceptual strength, relevance, and methodological rigor (including method of sample selection, data collection, data analysis, and statement of findings) (Kuper, Lingard, & Levinson, [23]), as well as overall contribution to existing knowledge or understanding [22].

Since the proposed review does not include a meta-analysis, this protocol could not be registered with PROSPERO.

#### Data synthesis

Since we anticipate both qualitative and quantitative studies as being eligible for this review, it is unlikely that available data will permit meta-analysis and assessment of treatment effects. To synthesize quantitative data, we will first prepare tabular summaries of the included studies’ characteristics. We will then appraise the data by type of design (including quality of design and methods); objectives; participants and settings; assessed outcomes; findings (including category, and correlates of barriers identified); and strengths, limitations, and value to existing body of knowledge. Guided by the review’s objectives and assessed outcome measures, we will synthesize qualitative data using the framework method of evidence synthesis for the systematic review of qualitative evidence. Details of this approach have been described elsewhere [24, 25]. Data synthesis will also include an examination of gaps in evidence and potential directions for research to address such gaps.

## Discussion

Despite the proliferation of MMLs, many patients with qualifying medical conditions lack access to medical marijuana. This review will identify barriers to medical marijuana among individuals living in states and territories with MMLs in the USA. This review will employ the structural violence framework to identify extrinsic factors that may serve as barriers to medical marijuana among patients with underlying conditions [26]. It is hoped that the structural violence framework will help bridge the gap between the proliferation of MMLs and lack of access to medical marijuana. Details on specific barriers to medical marijuana use will inform policymakers, clinicians, and patient advocates in efforts to eliminate these highlighted barriers.

## Appendix 1

**Table 1:** PRISMA-P (Preferred Reporting Items for Systematic review and Meta-Analysis Protocols) 2015 checklist: recommended items to address in a systematic review protocol

Section and topic	Item No.	Checklist item
ADMINISTRATIVE INFORMATION		
Title:		
Identification	1a	Identify the report as a protocol of a systematic review
Update	1b	If the protocol is for an update of a previous systematic review, identify as such
Registration	2	If registered, provide the name of the registry (such as PROSPERO) and registration number
Authors:		
Contact	3a	Provide name, institutional affiliation, e-mail address of all protocol authors; provide physical mailing address of corresponding author
Contributions	3b	Describe contributions of protocol authors and identify the guarantor of the review
Amendments	4	If the protocol represents an amendment of a previously completed or published protocol, identify as such and list changes; otherwise, state plan for documenting important protocol amendments
Support:		
Sources	5a	Indicate sources of financial or other support for the review
Sponsor	5b	Provide name for the review funder and/or sponsor
Role of sponsor or funder	5c	Describe roles of funder(s), sponsor(s), and/or institution(s), if any, in developing the protocol
INTRODUCTION		
Rationale	6	Describe the rationale for the review in the context of what is already known
Objectives	7	Provide an explicit statement of the question(s) the review will address with reference to participants, interventions, comparators, and outcomes (PICO)
METHODS		
Eligibility criteria	8	Specify the study characteristics (such as PICO, study design, setting, time frame) and report characteristics (such as years considered, language, publication status) to be used as criteria for eligibility for the review
Information sources	9	Describe all intended information sources (such as electronic databases, contact with study authors, trial registers or other gray literature sources) with planned dates of coverage
Search strategy	10	Present draft of search strategy to be used for at least one electronic database, including planned limits, such that it could be repeated
Study records:		
Data management	11a	Describe the mechanism(s) that will be used to manage records and data throughout the review
Selection process	11b	State the process that will be used for selecting studies (such as two independent reviewers) through each phase of the review (that is, screening, eligibility and inclusion in meta-analysis)
Data collection process	11c	Describe planned method of extracting data from reports (such as piloting forms, done independently, in duplicate), any processes for obtaining and confirming data from investigators
Data items	12	List and define all variables for which data will be sought (such as PICO items, funding sources), any pre-planned data assumptions and simplifications
Outcomes and prioritization	13	List and define all outcomes for which data will be sought, including prioritization of main and additional outcomes, with rationale
Risk of bias in individual studies	14	Describe anticipated methods for assessing risk of bias of individual studies, including whether this will be done at the outcome or study level, or both; state how this information will be used in data synthesis
Data synthesis	15a	Describe criteria under which study data will be quantitatively synthesized
	15b	If data are appropriate for quantitative synthesis, describe planned summary measures, methods of handling data and methods of combining data from studies, including any planned exploration of consistency (such as *I* ^2^, Kendall’s *τ*)
	15c	Describe any proposed additional analyses (such as sensitivity or subgroup analyses, meta-regression)
	15d	If quantitative synthesis is not appropriate, describe the type of summary planned
Meta-bias(es)	16	Specify any planned assessment of meta-bias(es) (such as publication bias across studies, selective reporting within studies)
Confidence in cumulative evidence	17	Describe how the strength of the body of evidence will be assessed (such as GRADE)

It is strongly recommended that this checklist be read in conjunction with the PRISMA-P Explanation and Elaboration (cite when available) for important clarification on the items. Amendments to a review protocol should be tracked and dated. The copyright for PRISMA-P (including checklist) is held by the PRISMA-P Group and is distributed under a Creative Commons Attribution Licence 4.0

From: Shamseer et al. [27]

## Appendix 2

### Search strategy

**PUBMED**

**Table 2:** Pubmed

Search #	Search Strategy
1	“structural barriers”[all fields] OR “Structure near2 Barrier” OR “Cultural Characteristics”[Mesh] OR “Cross-Cultural Comparison“[Mesh] OR “Hierarchy, Social”[Mesh] OR “Sociological Factors”[Mesh] OR “Social Marginalization”[Mesh] OR “Social near2 marginal*” OR “social norms”[Mesh] OR “Social near2 Norms” OR “social class”[Mesh] OR “Social near2 Class”
2	“Sexism”[mesh] OR “gender inequality”[all fields] OR “gender inequity”[all fields] OR “gender inequity”[all fields] OR “gender discrimination”[all fields] OR “Gender near2 Discriminat*” OR “racism”[Mesh] OR “minority stress”[all fields] OR “prejudice”[all fields] OR “white privilege”[all fields] OR “social perception”[Mesh] OR “self concept”[Mesh] OR “self-concept”[all fields] OR “social discrimination”[Mesh] OR “social disapproval”[all fields] OR (“perception”[Mesh] AND “social discriminat*”[Mesh]) OR “racial profiling”[all fields] OR “social conformity”[Mesh] OR “social bias”[all fields]
3	“Attitude to Health”[Mesh] OR “Health Knowledge, Attitudes, Practice”[Mesh] OR “Patient Dropouts”[Mesh] OR “Patient Participat*”[Mesh] OR “Patient Satisf*”[Mesh] OR “Patient near2 Satisfy*” OR “Patient Prefer*”[Mesh] OR “Patient near2 Prefer*” OR “Physician-Patient Relations”[Mesh] OR “Practice Patterns, Physicians'”[Mesh] OR “Medically Underserved Area”[Mesh] OR “Refus* to Treat”[Mesh] OR “Attitude of Health Personnel”[Mesh] OR “Attitude of Health Work*” OR “Refus* to Treat”[Mesh]
4	“poverty”[Mesh] OR “poverty areas”[Mesh] OR “medical indigency”[Mesh] OR “low income”[all fields] OR “low-income”[all fields] OR “Socioeconomic Factors”[Mesh] OR “low SES”[all fields] OR “unemploy*”[all fields] OR “under employed”[all fields] OR “under-employed”[all fields] OR “indigent”[all fields] OR “indigen*”[all fields] OR “uninsur* patients”[all fields] OR “uninsur* patient”[all fields] OR “cost of ill*”[Mesh] OR “health expen*”[Mesh]
5	“stigma*”[all fields] OR “social stigma”[Mesh] OR “Moral Development”[Mesh] OR “stereotypes”[all fields] OR “stereotyping”[Mesh]
6	“Medical Marijuana”[Mesh] OR “medical cannabis”[tiab] OR “medical marijuana”[tiab]

**Web of Science**

**Table 3:** Web of Science

#2	TS = (poverty) OR TS = (poverty areas) OR TS = (medical indigen*) OR TS = (low income) OR TS = (low-income) OR TS = (Socioeconomic Factors) OR TS = (low SES) OR TS = (unemploy*) OR TS = (under employ*) OR TS = (under-employ*) OR TS = (indigent) OR TS = (indigency) OR TS = (uninsured patients) OR TS = (uninsured patient) OR TS = (cost of illness) OR TS = (health expen*)
#3	TS = (Attitude to Health) OR TS = (Health Knowledge, Attitudes, Practice) OR TS = (Patient Dropouts) OR TS = (Patient Participat*) OR TS = (Patient Satisfaction) OR TS = (Patient Preference) OR TS = (Physician-Patient Relations) OR TS = (Practice Patterns, Physicians') OR TS = (Medically Underserved Area) OR TS = (Refusal to Treat) OR TS = (Attitude of Health Personnel) OR TS = (Refus* to Treat)
#4	TS = (Sexism) OR TS = (gender inequality) OR TS = (gender inequity) OR TS = (gender inequity) OR TS = (gender discriminat*) OR TS = (racism) OR TS = (minority stress) OR TS = (prejudice) OR TS = (white privilege) OR TS = (social perception) OR TS = (self concept) OR TS = (self-concept) OR TS = (social discriminat*) OR TS = (social disapproval) OR TS = (perception AND social discriminat*) OR TS = (racial profiling) OR TS = (social conformity) OR TS = (social bias)
#5	TS = (structural barriers) OR TS = (Cultural Characteristics) OR TS = (Cross-Cultural Comparison) OR TS = (Hierarchy, Social) OR TS = (Sociological Factors) OR TS = (Social Marginalization) OR TS = (social norms) OR TS = (social class)

**CINAHL**

**Table 4:** EbscoHOST CINAHL

#1	(MH “stigma”) OR “social stigma*” OR “stereotypes” OR (MH “stereot*”)
#2	(MH “poverty”) OR (MH “poverty areas”) OR “low income” OR (MH “Socioeconomic Factors”) OR "low SES" OR (MH “unemploy*”) OR “under employ*” OR (MH “indigent persons“OR (MH “medically uninsured”) OR "uninsured patients" OR (MH “economic aspects of illness”} OR “cost of illness” OR “health expen*”
#3	(MH “Attitude to Health”) OR (MH “Health Knowledge”) OR “Patient Dropouts” OR “Patient Participat*” OR (MH “Patient Satisf*”) OR "patient preference" OR (MH “Physician-Patient Relations”) OR (MH “Practice Patterns) OR (MH “Medically Underserved Area”) OR (MH “Refus* to Treat*”) OR (MH “Attitude of Health Personnel”)
#4	(MH “sex factor) OR (MH “race factors) OR (MH “Sexism”) OR (MH “gender bias”) OR “gender inequality” OR “gender inequity” OR “gender discriminat*” OR (MH “racism”) OR “minority stress” OR (MH “discriminat*”) OR (MH “prejudice”) OR “white privilege” OR (MH “social attitudes”) OR “social perception” OR (MH “self concept) OR “self-concept” OR “social discriminat*” OR “social disapproval” OR (MH “perception”) AND (MH “discrimination”) OR “racial profiling” OR “racial factors” OR (MH “social conform*”) OR “social bias”
#5	“structural barriers” OR “Cultural Characteristics” OR “Cross-Cultural Comparison“OR (MH “social environment”) OR “social hierarchy” OR (MH “social isolation”) OR “Sociological Factors” OR (“Social Marginal*”) OR (MH “social norms”) OR (MH “social class”)

**MEDLINE**

**Table 5:** MEDLINE

#1	stigma.mp. or exp social stigma/or exp Moral Development/or stereotypes.mp. or exp stereotyping/
#2	exp poverty/or exp poverty areas/or exp medical indigency/or low income.mp. or low-income.mp. or exp Socioeconomic Factors/or low SES.mp. or unemployment.mp. or under employed.mp. or under-employed.mp. or indigent.mp. or indigency.mp. or uninsured patients.mp. or uninsured patient.mp. or exp cost of illness/or exp health expenditures/
#3	exp Attitude to Health/or exp Health Knowledge, Attitudes, Practice/or exp Patient Dropouts/or exp Patient Participation/or exp Patient Satisfaction/or exp Patient Preference/or exp Physician-Patient Relations/or exp Practice Patterns, Physicians/or exp Medically Underserved Area/or exp Refusal to Treat/or exp Attitude of Health Personnel/or exp Refusal to Treat/
#4	exp Sexism/or gender inequality.mp. or gender inequity.mp. or gender inequity.mp. or gender discrimination.mp. or racism.mp. or minority stress.mp. or prejudice.mp. or white privilege.mp. or exp social perception/or exp self concept/or self-concept.mp. or exp social discrimination/or social disapproval.mp. or (exp perception/and exp social discrimination/) or racial profiling.mp. or exp social conformity/or social bias.mp. [mp = title, abstract, original title, name of substance word, subject heading word, keyword heading word, protocol supplementary concept word, rare disease supplementary concept word, unique identifier]
#5	structural barriers.mp. or exp Cultural Characteristics/or exp Cross-Cultural Comparison/or exp Hierarchy, Social/or exp Sociological Factors/or exp Social Marginalization/or exp social norms/or exp social class/
#6	exp medical marijuana/or medical marijuana.mp. or medical cannabis.mp.

**EMBASE**

**Table 6:** EMBASE

#1	‘stigma’/exp OR ‘social stigma’/de OR ‘morality’/exp OR ‘stereotypy’/exp OR ‘stereotyping’/exp
#2	‘poverty’/exp OR ‘poverty areas’ OR ‘medical indigency’ OR ‘low income’/exp OR ‘lowest income group’/exp OR’Socioeconomic’/exp OR ‘low SES’ OR 'unemploy*'/exp OR ‘under employ*’ OR ‘under-employ*’ OR 'indigent'/exp OR ‘indigency’ OR ‘medic* uninsur*’/exp OR ‘uninsured patients’ OR ‘uninsured patient’ OR ‘cost of ill*’/exp OR ‘health care cost’/exp
#3	‘attitude to health’/exp OR ‘patient dropouts’/exp OR ‘patient participat*’/exp OR ‘patient satisfy*’/exp OR ‘patient preference’/exp OR ‘doctor patient relation’/exp OR ‘clinical practice’/exp OR ‘Medically Underserved Area’ OR ‘physician attitude’/exp OR ‘health personnel attitude’/exp OR ‘patient abandonment’/exp OR ‘Refusal to Treat’
#4	‘Sexism’/exp OR ‘gender bias’/exp OR ‘gender inequality’ OR ‘gender inequity’ OR ‘gender discriminat*’ OR ‘racism’/exp OR ‘minority stress’ OR ‘prejudic*’ OR ‘white privilege’ OR ‘perception’/exp OR ‘social perception’ OR ‘self concept’/exp OR ‘self-concept’ OR ‘social discriminat*’/exp OR ‘social disapproval’ OR ‘perceptive discriminat*’/exp OR ‘racial profil*’ OR ‘social psychology’ OR ‘social conformity’ OR ‘social bias’
#5	‘structural barriers’ OR ‘structure near2 barrier’ OR ‘ethnic or racial aspects’/exp OR ‘cultural factor’/exp OR ‘social dominance’/exp OR ‘social aspects and related phenomen*’/exp OR ‘social exclu*’/exp OR ‘Social Marginal*’ OR ‘social norms’ OR ‘social norm’/exp OR ‘social class’/exp

**PsycINFO**

**Table 7:** EbscoHost PsycINFO

S1	DE “Stigma” OR TX “social stigma” OR DE “Stereotyped Attitudes” OR TX “stereotypes” OR TX “stereotyping” DE “Labeling”
S2	DE “poverty” OR DE poverty areas OR TX medical indigency OR TX “low income” OR DE “Socioeconomic status” OR TX “Socioeconomic Factors” OR TX “low SES” OR DE “lower income level” OR DE “unemployment” OR DE “under employed” OR DE “disadvantaged” OR DE “indigent” OR TX “indigency” OR TX “Medically Underserved Areas” OR DE “uninsured (health insurance)” OR DE “underinsured (health insurance)” OR TX “uninsured patient” OR DE “health care cost”
S3	DE” health attitudes” OR DE “Drug usage attitudes” OR DE “client Participation” OR DE “client Satisfaction” OR DE “client attitudes” OR DE “clinical practice” OR TX “Refusal to Treat” OR DE “Health Personnel Attitudes OR DE “treatment barriers” OR DE “treatment dropouts”
S4	DE “Sex Role Attitudes” OR TX “Sexism” OR DE “gender equality” OR TX “gender inequality” OR TX “gender inequity” OR TX “gender discrimination” OR DE “racism” OR DE “social stress” OR TX “minority stress” OR DE “prejudice” OR TX “white privilege” OR DE “social perception” OR DE “self-concept” OR DE “social discrimination” OR TX “social disapproval” OR (DE “perception” AND “social discrimination”) OR DE “criminal profiling” OR TX “racial profiling” OR TX “social conformity” OR TX “social bias”
S5	TX “structural barriers” OR DE “Cross cultural treatment” OR DE “Cross Cultural Differences“OR DE “Dominance Hierarchy” OR DE “sociocultural factors” OR TX “Social Marginalization” OR DE “Marginalization” OR DE “Social Acceptance” OR DE “social approval” OR DE “social norms” OR DE “Social issues” OR DE “social class”

## Abbreviation

MML: Medical marijuana laws

## References

[1] Substance Abuse and Mental Health Services Administration (SAMHSA). Results from the 2012 National Survey on drug use and health: summary of national findings, NSDUH Series H-46, HHS Publication No. (SMA) 13–4795. Rockville: Substance Abuse and Mental Health Services Administration; 2013. Available from: http://www.samhsa.gov/data/NSDUH/2012SummNatFindDetTables/NationalFindings/NSDUHresults2012.htm. Accessed 22 July 2017.

[2] Pew Research Center. Majority now supports legalizing marijuana. 2013. Available from: http://www.people-press.org/2013/04/04/majority-now-supports-legalizing-marijuana. Accessed 22 July 2017.

[3] Desai U, Patel P (2013). Medical marijuana: a public health perspective.

[4] NORML., (no date). States that have decriminalized medical marijuana. Available from: http://norml.org/aboutmarijuana/item/states-that-have-decriminalized. Accessed 22 July 2017.

[5] Cerdá M, Wall M, Keyes KM, Galea S, Hasin D. Medical marijuana laws in 50 states: investigating the relationship between state legalization of medical marijuana and marijuana use, abuse and dependence. Drug Alcohol Depend. 2012;120(1):22–7. 10.1016/j.drugalcdep.2011.06.011 PMC3251168 22099393

[6] Farmer PE, Nizeye B, Stulac S, Keshavjee S (2006). Structural violence and clinical medicine. PLoS Med.

[7] Shaw D (2008). Social determinants of health. Clin Med.

[8] Friesthler B, Gruenewald PJ (2014). Examining the relationship between the physical availability of medical marijuana and marijuana use across fifty California cities. Drug Alcohol Depend.

[9] Richmond MK, Pampel FC, Rivera LS, Broderick KB, Reimann B, Fischer L (2015). Frequency and risk of marijuana use among substance-using health care patients in Colorado with and without access to state legalized medical marijuana. J Psychoactive Drugs.

[10] Reinarman C, Nunberg H, Lanthier F, Heddleston T (2011). Who are medical marijuana patients? Population characteristics from nine California assessment clinics. J Psychoactive Drugs.

[11] Menjívar C (2006). Liminal legality: Salvadoran and Guatemalan immigrants’ lives in the United States 1. Am J Sociol.

[12] Kochhar R, Fry R. Wealth inequality has widened along racial, ethnic lines since end of Great Recession. Pew Res Center. 2014;12.

[13] Chu YWL (2014). The effects of medical marijuana laws on illegal marijuana use. J Health Econ.

[14] Arizona Department of Health Services (ADHS). Medical Marijuana Program. 2015. Available from http://www.azdhs.gov/documents/licensing/medical-marijuana/reports/2015/2015-mm-annual-report-160114.pdf. Accessed 22 July 2017.

[15] Satterlund TD, Lee JP, Moore RS (2015). Stigma among California’s medical marijuana patients. J Psychoactive Drugs.

[16] Pacula RL, Powell D, Heaton P, Sevigny EL (2015). Assessing the Effects of Medical Marijuana Laws on Marijuana Use: The Devil is in the Details. J Policy Anal Manage.

[17] Michalec B, Rapp L (2014). Medical marijuana: the importance of education and research. Del Med J.

[18] Arksey H, O'Malley L (2005). Scoping studies: towards a methodological framework. Int J Social Res Methodol.

[19] Higgins JPT, Green S, editors. Cochrane Handbook for Systematic Reviews of Interventions Version 5.1.0 [updated March 2011]. The Cochrane Collaboration. 2011. Available from www.handbook.cochrane.org. Accessed 22 July 2017.

[20] Wells G, Shea B, O’Connell D, Welch V, Losos M, Tugwell P (2000). The Newcastle Ottawa Scale (NOS) for assessing the quality of nonrandomized studies in meta-analyses. Proceedings of the 3rd symposium on systematic reviews: beyond the basics.

[21] National Collaborating Centre for Environmental Health. A primer for evaluating the quality of studies on environmental health critical appraisal of cross-sectional studies. 2011. [cited 2013 Nov 19]. Available from: http://www.ncceh.ca/sites/default/files/Critical_Appraisal_Cross-Sectional_Studies_Aug_2011.pdf. Accessed 22 July 2017.

[22] Critical Appraisal Skills Programme (2017). CASP Qualitative Research Checklist.

[23] Kuper A, Lingard L, Levinson W. Critically appraising qualitative research. BMJ. 2008;337(aug073):a1035–a1035. 10.1136/bmj.a1035 18687726

[24] Carroll C, Booth A, Cooper K (2011). A worked example of “best fit” framework synthesis: a systematic review of views concerning the taking of some potential chemopreventive agents. BMC Med Res Methodol.

[25] Dixon-Woods M (2011). Using framework-based synthesis for conducting reviews of qualitative studies. BMC Med.

[26] Farmer P, Bourgois P, Scheper Hughes N, Fassin D, Green L, Heggenhougen HK, … Farmer P. An anthropology of structural violence 1. Curr Anthropol. 2004;45(3):305–25.

[27] Shamseer L, Moher D, Clarke M, Ghersi D, Liberati A, Petticrew M, … Stewart LA, PRISMA-P Group. Preferred reporting items for systematic review and meta-analysis protocols (PRISMA-P) 2015: elaboration and explanation. BMJ. 2015;349:g7647. 10.1136/bmj.g7647 25555855

